# ChipletQuake: On-Die Digital Impedance Sensing for Chiplet and Interposer Verification

**DOI:** 10.3390/s25154861

**Published:** 2025-08-07

**Authors:** Saleh Khalaj Monfared, Maryam Saadat Safa, Shahin Tajik

**Affiliations:** Worcester Polytechnic Institute, Worcester, MA 01609, USA; skmonfared@wpi.edu (S.K.M.); msafa@wpi.edu (M.S.S.)

**Keywords:** hardware security, hardware Trojans, power delivery network, tamper detection, chiplet security

## Abstract

The increasing complexity and cost of manufacturing monolithic chips have driven the semiconductor industry toward chiplet-based designs, where smaller, modular chiplets are integrated onto a single interposer. While chiplet architectures offer significant advantages, such as improved yields, design flexibility, and cost efficiency, they introduce new security challenges in the horizontal hardware manufacturing supply chain. These challenges include risks of hardware Trojans, cross-die side-channel and fault injection attacks, probing of chiplet interfaces, and intellectual property theft. To address these concerns, this paper presents ChipletQuake, a novel on-chiplet framework for verifying the physical security and integrity of adjacent chiplets during the post-silicon stage. By sensing the impedance of the power delivery network (PDN) of the system, ChipletQuake detects tamper events in the interposer and neighboring chiplets without requiring any direct signal interface or additional hardware components. Fully compatible with the digital resources of FPGA-based chiplets, this framework demonstrates the ability to identify the insertion of passive and subtle malicious circuits, providing an effective solution to enhance the security of chiplet-based systems. To validate our claims, we showcase how our framework detects hardware Trojans and interposer tampering.

## 1. Introduction

The increasing complexity of monolithic chips has led to skyrocketing costs and significant technical challenges over the last decade. As semiconductor manufacturers push toward smaller nodes, the sophistication of scaling down components while maintaining performance, power efficiency, and cost becomes more difficult. The need for advanced lithography, increased heat dissipation, and managing yield issues from larger die sizes are driving up costs exponentially. On the other hand, manufacturing large-scale Systems-on-Chip (SoCs) brings significant challenges regarding post-silicon testing procedures. In response, the industry has shifted toward multi-chip module (MCM) designs, where multiple smaller chiplets are integrated onto a single interposer. Such heterogeneous packaging approaches offer several advantages, such as allowing the combination of different process technologies [1]; improving yields by using smaller, modular dies; and enhancing scalability [1,2]. Additionally, chiplet designs offer better flexibility, allowing companies to reuse proven designs and mix-and-match chiplets to create custom solutions, significantly reducing both development time and cost [3].

**Figure 1:**
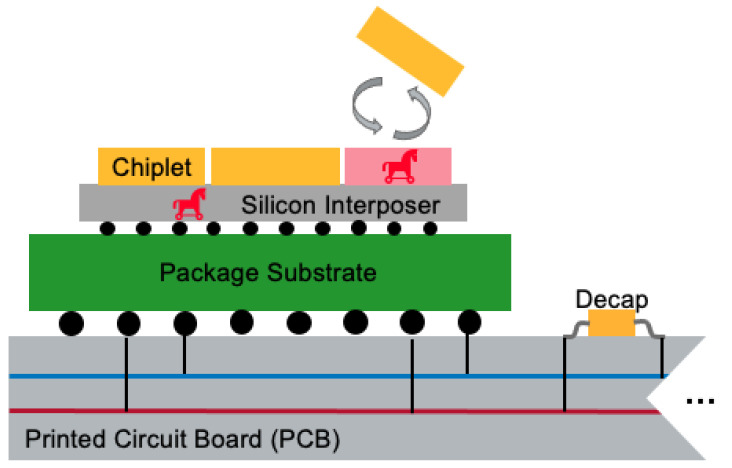
Threats in chiplet-enabled systems include die-swapping, susceptibility to interposer probing, and tampering.

Contrary to monolithic ICs, creating systems from separately produced components creates security issues, e.g., the possibility of die-swapping, susceptibility to interposer probing, or tampering, specifically in black-boxthreat models (see Figure 1). In a zero-trust security model, a chiplet should be able to verify the host interposer and other chiplets. Verification schemes such as delay-based Physical Unclonable Functions (PUFs) between chiplets have been developed, where the signal propagation delays through the interposer are considered fingerprints [4]. However, there might be no direct signal connection between the verifier and prover chiplets to realize such PUFs. Moreover, there could be sophisticated probing or tampering attacks on chiplet interfaces [5], which enable direct eavesdropping from the interposer wires.

**Figure 2:**
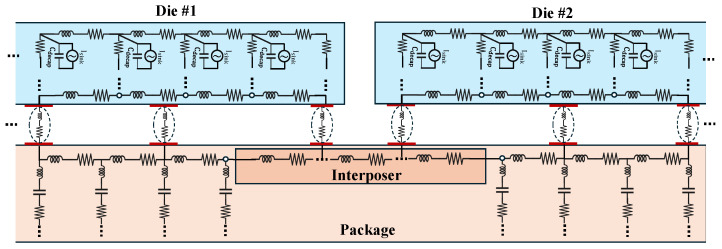
A simple circuit model of a PDN and interposer, inspired by [10]. Note that dashed ovals represent the equivalent interposer impedance.

In MCMs, multiple chiplets and interposers are interconnected through a shared power delivery network (PDN). Figure 2 shows a simple model of a PDN and interposer. The PDN distributes power from a central source to each chiplet. There have been a few attempts in the literature [6] to include self-contained sensors on one of the chiplets to detect anomalies in adjacent chiplets. On digital ICs and Field-Programmable Gate Arrays (FPGAs), these sensors take the form of delay-based circuits such as on-chip ring oscillators (ROs) and time-to-digital converters. If all goes well, any anomalies in running applications will then affect the sensitive timing behavior of these sensors. However, such passive sensing methods have led to low-precision measurements and noisy behavior. Therefore, advanced machine learning methods are needed to obtain acceptable classification accuracy. Moreover, all these solutions demonstrate only the detection of specific anomaly behavior in running software; they are not applicable to physical tamper events, such as dormant hardware Trojans, nor have they been applied to extract static information [7].

On the other hand, PDN-based impedance signatures have been identified as potential attack vectors for chiplet fingerprinting in multi-tenant cloud FPGAs [8]. Existing impedance analysis methods are only able to cover a limited frequency bandwidth, which limits their ability to extract intra-chip characterization. Although recent research [9] suggests that high-frequency impedance analysis offers high-quality signals for extracting transistor-level information, inter-die and interposer impedance characterization have yet to be studied in the context of hardware verification and side-channel leakage for chiplets.

**Research Questions**

In line with recent trends in PDN-based profiling, the following research questions identify key gaps in the current understanding of chiplet-based side channels. Addressing these questions constitutes the ultimate goal of this paper:

1. Does there exist a sensor on a trusted chiplet that can physically verify its environment beyond itself, from the interposer to neighboring chiplets, in a unified manner?
2. Considering that existing power-based side channels, which only monitor dynamic power activities, can impedance side channels provide high SNR capable of detecting tiny static transistor-level information with a focus on dormant HTs?
3. Is high-frequency impedance sensing feasible without requiring additional analog elements and interfaces on chiplets?

In this article, we show that circuit-level elements (in terms of their hardware placements and routing) can potentially be fingerprinted in a PDN-shared heterogeneous system with multiple chiplets in a single package. Our method relies on the fact that physical modifications (regardless of their physical size, activation, or action characteristics) alter the impedance of the shared PDN in the frequency domain. Therefore, characterizing the impedance at high frequencies can facilitate the extraction of information about the hardware state of chiplets. Furthermore, we aim to demonstrate that such impedance signatures can serve as reliable identifiers for chiplet verification and anomaly detection in heterogeneous multi-chiplet systems.

**Contributions**

For this purpose, we present a fully digital framework that systematically performs a frequency-sweeping mechanism to estimate the impedance that can identify the integrity of the internal circuitry of the chiplets and the corresponding interposer interconnection circuits. In summary, to address the primary objectives outlined in this work, the key contributions of this manuscript are as follows:

- This article is the first work to introduce a fully digital and spectral-assisted method for accurately ensuring the physical integrity of chiplets.
- We design a framework called ChipletQuake, which provides efficient and effective hardware verification of adjacent chiplets and the host interposer.
- We implement ChipletQuake on a high-end chiplet-based FPGA system and perform extensive experiments against various hardware Trojans, showcasing the applicability of our framework.

## 2. Technical Background

### 2.1. Power Delivery Network

A PDN is critical in providing a stable and low-noise voltage supply to the electronic components on a PCB, spanning from the voltage regulator module (VRM) to the power rails on the chip. The PDN is composed of both off-chip and on-chip elements, including bulk capacitors, PCB routing, ceramic capacitors, PCB planes, vias, package bumps, on-chip power planes, and transistor capacitance. In the case of multi-die systems, the interposer and its corresponding μbumps and inter-die interconnections are also part of the PDN. Each of these components contributes to the PDN’s impedance across different frequency regimes.

At low frequencies, the impedance of the PDN is primarily governed by the voltage regulator and off-chip components. As the frequency increases, the impedance behavior changes significantly, with the on-chip components contributing more to the PDN impedance at higher frequencies. This shift is largely due to the parasitic inductance inherent in each capacitor, which affects their behavior at different frequencies. At higher frequencies, capacitors exhibit a resonance phenomenon caused by the parasitic inductance in their metals. Beyond this resonance frequency, the capacitors behave like open circuits, drastically reducing their impact on the PDN’s impedance. Smaller capacitors, with lower parasitic inductance, resonate at higher frequencies, meaning their influence on the PDN impedance diminishes as the frequency increases. Consequently, at very high frequencies, the impedance is dominated by interposer and on-chip structures, which are characterized by smaller dimensions.

**Figure 3:**
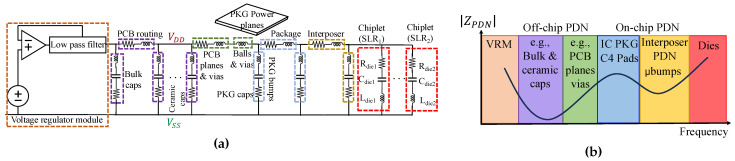
(**a**) Equivalent RLC circuit model of the power distribution network of the PCB and chip. (**b**) Contribution of different parts of the PDN to the impedance over frequency [11].

The on-chip PDN behavior is modeled using an equivalent RC circuit, where the on-chip capacitance is represented by multiple narrow-band parallel RC circuits connected to the VDD and VSS power rails (see Figure 3). These circuits allow for an accurate representation of the PDN impedance over a wide frequency range. The impedance characteristics of the PDN in the frequency domain provide valuable insights into the system’s behavior and can even be used to detect tampering events at the PCB level. Tampering with components inside the integrated circuit (IC), such as altering logic gates, placement, or routing, can change the on-chip capacitance, which, in turn, affects the PDN’s impedance. This impact depends on the size, location, and nature of the tampering and demonstrates the sensitivity of the PDN to modifications within the chip [11].

### 2.2. Interposer Power Distribution Network

Today’s PDN design for multi-chip modules is meticulously crafted to manage the high power demands of large-scale compute circuits while maintaining signal integrity and effective thermal management. In this work, we focus on Xilinx/AMD’s Stacked Silicon Interconnect (SSI) technology, which provides chiplet-based interconnections in multi-chip FPGAs. At the core of this architecture is the silicon interposer, a passive layer that facilitates routing for configuration, global clocking, and interconnect signals between the programmable logic units known as Xilinx’s Super Logic Regions (SLRs). Each SLR in the SSI-enabled device operates with independent power delivery, with power and ground connections routed through the interposer. These connections are facilitated by Through-Silicon Vias (TSVs), which create low-resistance paths between the active layers of the FPGA and the package substrate. This segmentation minimizes cross-talk between regions, enhancing both power delivery efficiency and overall signal integrity.

The interposer enables the use of separate power planes for core logic, I/O, transceivers, and memory interfaces, reducing electrical noise and ensuring the stable operation of sensitive circuitry. Furthermore, it distributes power uniformly across the SLRs and facilitates clocking and configuration connections while also contributing to thermal management. At the chip level, the PDN forms a complex passive network that delivers power to each computing unit via the silicon interposer. The power supply transitions through interconnects between the package and the interposer layer before being locally distributed by the die power grid, an irregular, multi-layer metal mesh that connects to lower-level digital circuits.

### 2.3. Coupling Effect Between SLRs

The coupling effect in 2.5D integrated systems occurs due to shared resources, such as the PDN and interposer, which interconnect multiple SLRs. This effect is driven by both power and electromagnetic interactions. Variations in power consumption by one SLR induce fluctuations in the shared PDN, creating voltage and current perturbations that propagate across the interposer and impact neighboring SLRs. These fluctuations result from the resistive, capacitive, and inductive characteristics of the PDN, including the mutual inductance and parasitic capacitance of vias and traces, as well as in the closely spaced power and ground planes. Additionally, the switching activity in one SLR generates alternating electric and magnetic fields that can couple into adjacent SLRs through electromagnetic interference. These electromagnetic coupling signatures, while enabling SLR interaction and optimization, also serve as reliable indicators for tamper detection and system security by revealing anomalies in power or electromagnetic behaviors [12].

### 2.4. Delay-Based On-Chip Sensor

It has been demonstrated in multiple cases that voltage fluctuations resulting from computation on the IC’s die can be measured using delay-based circuits placed on the same die [13]. Power consumption alteration affects the propagation delays of electrical signals. Such a change in propagation delay can be recorded in on-die sensory circuits, such as ring oscillators [14] and time-to-digital converters (TDCs) [13]. These sensors have been used to detect voltage, electromagnetic (EM), and laser glitching and radiating attacks on FPGAs [15,16]. However, they have not been used for integrity checks on multi-chip or chiplet systems. The implementation of RO-based sensors incurs high power usage, and they perform poorly for accurate sensing (with picosecond granularity). Therefore, in this paper, we utilize TDC sensors for our integrity-checking framework.

**Figure 4:**
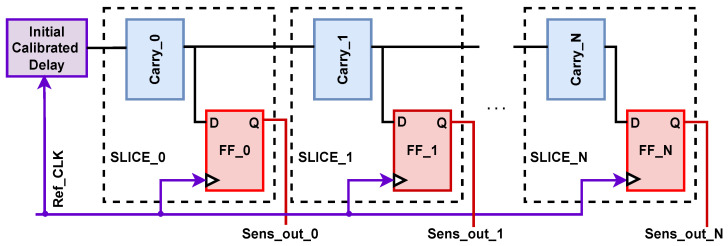
High-level implementation of a TDC-based fault detection sensor.

In this work, inspired by the TDC implementation described by Gnad et al. [17], we re-purpose a TDC design for integrity-checking purposes on multi-die FPGAs. Figure 4 depicts a high-level implementation of the employed TDC. As highlighted, the sensor’s core components include an initial delayed signal, a tapped delay line, and corresponding output registers. The calibration process is necessary for the TDC sensors and is performed offline by adjusting the delay elements (e.g., CARRY8) or by modifying the chain of combinational logic blocks. This process ensures that the sensor’s output reaches a metastable state that is suitable for accurately sensing tiny voltage fluctuations. The delay line typically employs fast carry propagation logic, physically constrained to form a sequential delay chain. Each delay unit’s output is propagated to an output register clocked by the original signal, with the binary sensor output determined by the propagation delay at each register’s input. Ideally, external high-speed sampling circuitry is deployed to record the output of the TDC sensors. In this work, we organize a 2D mesh of TDC sensors on the verifier chiplet and perform high-speed sampling on all TDCs simultaneously.

## 3. ChipletQuake

Considering recent concerns regarding potential chiplet-based security threats [18], defense against such threats requires a runtime and accurate integrity-checking mechanism in place. Motivated by the capacity of shared PDNs in chiplet-enabled systems and existing research on inter-chiplet fingerprinting [8], we develop a digital monitoring system to sense the impedance fluctuations of the physical environment of the chiplets. We present ChipletQuake to detect tiny and passive alterations in the interposer, as well as neighboring chiplets, which can serve as a verification framework to identify hardware Trojans (HTs).

### 3.1. High-Level Design

**Figure 5:**
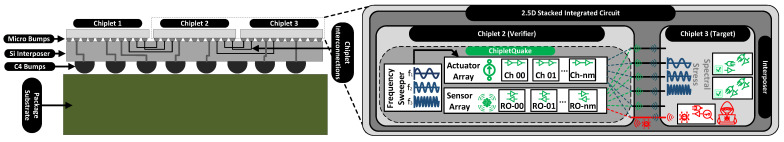
High-level overview of ChipletQuake functionality.

Figure 5 shows the high-level design of ChipletQuake. As shown, the verifier chiplet is equipped with some internal components of a monitoring system. Using a digitally designed frequency sweeper, an array of power-wasting circuity (e.g., inverter chains), referred to as the actuator array, is activated to oscillate at certain frequencies. Due to the shared PDN in the system, neighboring chiplets undergo frequency-dependent current and voltage fluctuations. At the same time, another sensory circuit in ChipletQuake records these fluctuations by accurately sensing the voltage via digital delay-based or ring oscillator-based sensors.

During the monitoring routine, if the verifier identifies any noticeable deviation from the valid existing measurement (golden model) of the sensed values, it considers a possible malicious hardware alteration in the neighboring chiplets or, as shown, on the interposer itself.

### 3.2. Chiplet Verification Flow

**Figure 6:**
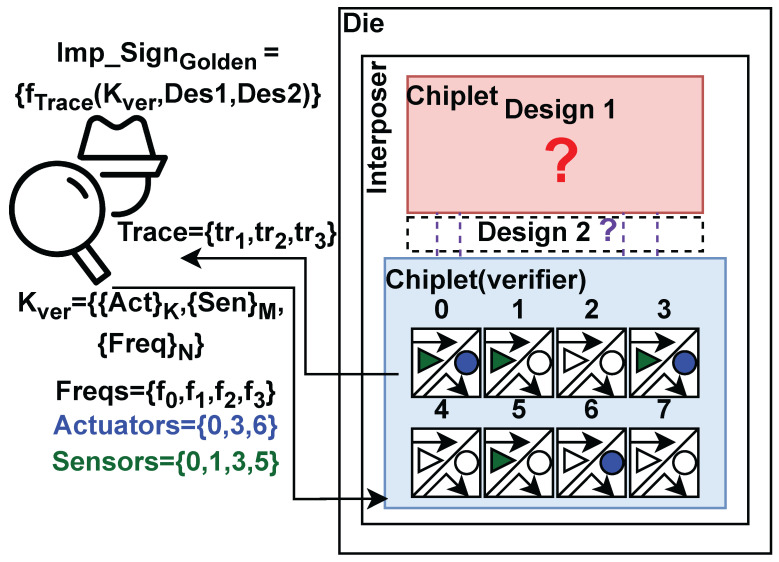
Chiplet verification flow via impedance estimation. Question marks indicate the components within the system that require verification. 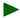 and 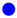 represent the sensors and actuators, respectively, within our framework.

To facilitate a reliable hardware-level integrity check, we develop a verification protocol that utilizes a challenge–response authentication scheme [19]. Instead of conventional digital signatures, a specific estimation of circuit impedance is considered the response (signature). As illustrated in Figure 6, the verifier chiplet deploys a 2D array on its reconfigurable fabric (e.g., FPGA). Inside each element of the array located in the chiplet layout, a sensor (e.g., TDC) is implemented, which is highlighted as a filled 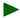, if enabled during the verification process.

Furthermore, elements of the arrays are also equipped with a current actuator, illustrated as a filled 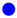, once activated. Consequently, the verifier organizes a 2D grid of sensors and power wasters.

To extract a proper impedance-based signature as the golden model, the verifier first chooses a set of *N* frequencies $\left\{Freq_{N}\right\}=\left\{f_{0},f_{1},f_{2},\ldots f_{N-1}\right\}$ in which the impedance is estimated during the verification process. Then, by choosing a set of corresponding IDs in the grid, *K* actuator elements are selected. For instance, in Figure 6, actuators from the regions $\left\{Actuators\right\}=\left\{AC_{0},AC_{3},AC_{6}\right\}$ are selected and highlighted. These actuators are then activated and fed with the selected frequencies from the $\left\{Freq_{N}\right\}$ set. At the same time, the verifier selects another set of *M* IDs, which are associated with the sensors that are responsible for recording the voltage-based fluctuations. As a simple example, in Figure 6, $\left\{Sensors\right\}=\left\{S_{0},S_{1},S_{3},S_{5}\right\}$ are chosen.

Performing the entire verification process locally on the verifier chiplet requires the verifier to store all the golden signatures on the verifier chip. However, due to the possibility of storage limitations, the large size of multiple golden signatures makes this approach infeasible in some cases. Furthermore, to comply with the requirements of remote attestation scenarios [20], the integrity check should provide guarantees for remote users. In this case, a large attack surface should be considered. Specifically, replay attacks on the signatures can bypass the verification process. Furthermore, dynamic reconfiguration of the target chiplets can affect the extracted signatures captured by the verifier. To overcome such challenges, ChipletQuake provides a one-time authentication protocol by utilizing a one-time verification key $K_{ver}$ as the challenge for the target adjacent chiplets and the interposer itself. The verification key is generated by randomly selecting sets of IDs of the actuators, sensors, and target frequencies for the impedance sensing. Our experiments, aligned with previous studies, show that each combination of the $\left\{K_{ver}\right\}=\left\{{\left\{Act\right\}}_{K},{\left\{Sen\right\}}_{M},{\left\{Freq\right\}}_{N}\right\}$ yields a unique impedance profile, which then can be post-processed to represent a valid golden signature.

After recording multiple golden impedance signatures using different $\left\{K_{ver}\right\}$s, the target device in the field can be tested on demand. The traces extracted from the verification process can be easily validated remotely. Note that, during the test process, the traces extracted from the chip’s environment are a function dependent on $\left\{K_{ver}\right\}$ (which protects the security of the authentication protocol) and the physical layout and state of the hardware circuitry. Hence, any malicious hardware-level modification on the system-in-a-package can be detected by analyzing the captured traces.

### 3.3. Frequency Band Selection

A PCB’s power and ground planes form a cavity that can resonate at specific frequencies, occurring when the electrical dimensions of the PCB align with integer multiples of the electromagnetic wavelength. At these resonant frequencies, the impedance of the PCB becomes more sensitive due to the interaction of interconnect parasitic inductance, parasitic capacitance, and decoupling capacitance. This intensified sensitivity makes the PCB more susceptible to even small changes in the impedance, such as those caused by a hardware Trojan and tampering events. These small changes can introduce additional parasitics or alter signal pathways, potentially leading to observable performance anomalies.

Additionally, at resonant frequencies, electromagnetic energy tends to concentrate in certain regions of the PCB. This localized accumulation of energy creates areas where the presence of a hardware Trojan can have a significant impact. A Trojan embedded in such regions may interact with the concentrated electromagnetic fields, leading to detectable deviations in signal integrity, power delivery, or electromagnetic emissions [21]. The resonant frequency of a rectangular cavity resonator can be derived from the following equation:

(1) $$f_{r}=\frac{1}{2\pi \sqrt{\mu \epsilon }}\cdot \sqrt{{\left(\frac{m\pi }{a}\right)}^{2}+{\left(\frac{n\pi }{b}\right)}^{2}+{\left(\frac{p\pi }{d}\right)}^{2}}$$

where a and b are the cross-sectional sizes, d is the length of the cavity, and ϵ and μ are the permittivity and permeability of the material that fills the cavity, respectively [22].

Knowing the physical dimensions of the target PCB and its relative permittivity, the fundamental resonance frequencies can be estimated for measurements. In this work, we adopt a broad-frequency-range impedance sensing approach based on differential analysis, as detailed in [23]. To ensure coverage of potential resonance points, we sweep the frequency across a broad range. This selection is guided by the cavity resonance model described in prior works [8,23], which suggests that PCB and package dimensions result in frequency-dependent impedance sensitivity within this band. Although precise resonance peaks are not derived for each test platform, the broad frequency range is sufficient to capture relevant impedance fluctuations, enabling the detection of hardware-level anomalies.

### 3.4. Experimental Scope and Baseline Configuration

ChipletQuake introduces a flexible challenge–response framework with a large configuration space, allowing the arbitrary selection of actuator elements, sensor positions, and excitation frequencies for authentication and tamper detection. The experiments presented in this work focus on a baseline configuration to validate the core sensing methodology. Specifically, we deploy two TDC sensors and perform actuation across the entire available frequency band, using a linearly spaced set of frequency points that includes both low- and high-frequency components. This setup ensures that the measurements capture the resonance behaviors and impedance sensitivities discussed in Section 3.3. The goal of this baseline is to demonstrate the feasibility and statistical reliability of impedance-based sensing before scaling to more complex verification protocols.

### 3.5. Implementation Layout

**Figure 7:**
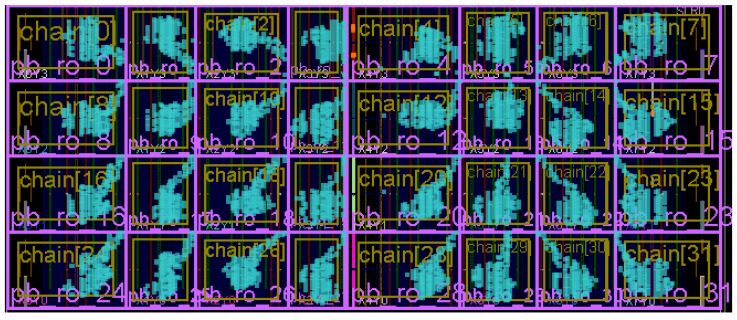
ChipletQuakeFPGA implementation layout with an array of 32 monitoring blocks.

To implement ChipletQuake, it is vital to properly carry out the physical placement of the underlying blocks. Specifically, we impose physical constraints on the chiplet’s fabric to ensure that the sensors and actuators are evenly distributed and deployed. Figure 7 shows an implementation layout of ChipletQuake with 32 monitoring blocks on an FPGA chiplet. As mentioned, each block comprises a TDC sensor and an inverter-based actuator. Separated physically, each of these elements is controlled individually by a controller and can be configured to operate in different modes. Specifically, the sampling rate of the sensors, input frequency of the actuator chain, and sensing time interval can be remotely configured as parameters during the verification procedure.

## 4. Case Studies

### 4.1. Threat Model

Modern chiplet architectures necessitate design teams to integrate or procure chiplet intellectual property (IP) from external vendors. However, it is impractical for these design teams to be directly involved in the development of every individual chiplet. Consequently, organizations that outsource chiplet components must depend on the manufacturers of these chiplets to deliver reliable hardware, making it essential to implement robust security measures. In such a horizontal supply chain, to guarantee the quality and integrity of the products, outsourcing companies should adopt a zero-trust security model where no inherent trust is granted to devices or users, the network environment, or ownership [24]. Applying a zero-trust approach requires the verification of all chiplet hardware, regardless of its source. Key vulnerabilities in chiplet-based systems include risks such as hardware tampering, unauthorized probing, and the insertion of hardware Trojans. Given the extensive and global nature of the chiplet supply chain, authentication is critical to ensure that each chiplet meets the required standards and specifications.

To address these challenges, our fingerprinting method ensures the authenticity of the adjacent chiplets and the host interposer by exploiting digital impedance estimation. Furthermore, existing authentication mechanisms depend on keeping the challenge–response pair secure from adversaries, which requires that the key itself be stored within a trusted environment. However, in the proposed method, the physical characteristics of the hardware account for the authenticity of the target hardware. Hence, any malicious tampering with the hardware yields an invalid key embedded in the impedance profile.

In our threat model, we consider a multi-chiplet system in which a single challenger chiplet verifies the integrity of the adjacent chiplet and the interposer. We assume no access to target chiplets in terms of logical challenge/response authentication. Moreover, our threat model requires the verifier to store post-silicon golden signatures prior to the test procedure. Furthermore, note that our threat model does not require the target chiplet circuitry to be activated in terms of transistor switching activity. This is a particularly useful model for detecting dormant and evasive hardware Trojans, which are often implemented via tiny units.

### 4.2. Experimental Scenarios

**Figure 8:**
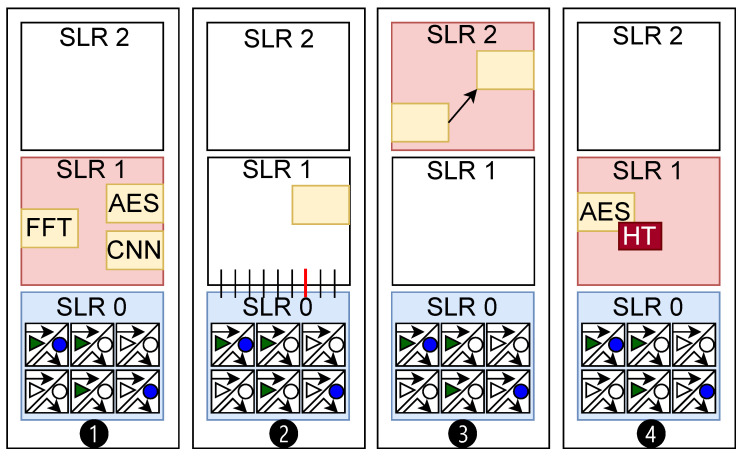
High-level illustrations of the evaluation case studies.

We organize different case studies to thoroughly investigate the detection capability of the proposed digital impedance sensing in chiplet-based FPGAs. In particular, in this work, we focus on four distinct case studies for chiplet verification. Figure 8 shows a high-level illustration of how each of these case studies is designed. As shown, in each of the scenarios, we deploy our verification hardware in chiplet-0 (SLR0) and perform verification for chiplets/interposers. We first study the case where different hardware modules are implemented in the adjacent SLR in ❶. Here, we capture the impedance estimation for three hardware implementations: Fast Fourier transform (FFT), Advanced Encryption Standard (AES), and Convolutional Neural Network (CNN). Then we investigate whether each of these hardware modules creates a distinguishable fingerprint that can be detected by our sensors in the SLR0.

In ❷, we study whether modifications on the host interposer can be effectively detected. For this purpose, we modify the utilization of communication lines between two chiplets. These lines are implemented and placed on the host interposer in the FPGA. The scenario here emulates attacks that include interposer tampering and probing. In ❸, we further investigate the sensitivity of our framework for distant chiplets. Specifically, we consider changing the placement of a single IP in SLR2 and monitoring the estimated profile on the verifier. Finally, in ❹, we investigate whether tiny hardware Trojans can be detected in adjacent chiplets.

## 5. Evaluation Results

### 5.1. Implementation Setup

For our evaluation, we used the Virtex™ UltraScale+™ VU37P HBM FPGA (Xilinx, Inc., San Jose, CA, USA) with 8GB of HBM DRAM memory. The target FPGA in this evaluation kit is the XCVU37P-L2FSVH2892E, which includes three programmable FPGA chiplets (i.e., SLRs) and a corresponding HBM DRAM chiplet. In our evaluations, we implemented ChipletQuake on SLR 0 as the verifier and performed the integrity/verification tests for other SLRs. Furthermore, the device was connected to a controller computer via a UART interface; the computer received the traces and generated the signature.

### 5.2. Implementation Details

For our ChipletQuake experiments, the actuators were implemented using chains of LUT1 inverters. The length of each chain could be adjusted to modulate the level of stimulation for impedance sensing. In our setup, each actuator element consisted of a chain of 10,000 inverters, with each chain driven by an adjustable PLL clock source that could be dynamically configured. Frequency sweeping was performed by custom-designed hardware within ChipletQuake, which down-sampled the PLL’s maximum frequency (MAX_FREQ) through digital synthesis and toggling mechanisms. For this set of experiments, the PLL’s MAX_FREQ was configured at 940 MHz. We employed an array of eight actuator elements for the impedance stimulation. For time-to-digital conversion (TDC), we used a 128-bit output width per sensor, implemented using 16 instances of the CARRY8 primitive. The TDC measurements were taken differentially: first, the actuator was activated, and the TDC sensor values were recorded while the PDN was under stimulation. Immediately afterward, the actuator was deactivated, and the TDC sensors were sampled again. The final TDC measurement was computed as the bitwise XOR of these two readings.

TDC measurements were integrated over each frequency point. The default integration time per frequency was 1 s. During this period, TDC outputs were sampled at the MAX_FREQ rate and averaged. For this evaluation, we used two TDC sensor elements placed on the verifier SLR. The frequency windows used in the ChipletQuake measurements were selected based on the experimental scenario. However, in most experiments, we swept across 20 or 50 frequency points linearly spanned within the 100 MHz to 900 MHz band for our impedance analysis.

### 5.3. Evaluation Metrics

As a commonly used metric in the hardware security domain, we chose Welch’s *t*-test [25] as the basic distance metric for our evaluations. For Welch’s *t*-test, small *p* values lead to the rejection of the null hypothesis of similar (normal) distributions in distinguishability tests. For the sake of simplicity, it is best practice to select a threshold of |t|>4.5 to reject the null hypothesis without considering the degree, to conclude that the sets were drawn from different populations [26].

Nevertheless, as the impedance profile distribution might not necessarily follow a Gaussian trend in some cases, we also included the distribution-agnostic Wasserstein metric [27] to carry out the distinguishability tests. The Wasserstein metric is the function that provides the distance between two probability distributions, each extracted from impedance estimation via ChipletQuake. The *p*th (p≥1) Wasserstein distance between $\gamma_{i}$ and $\tau_{i}$ is given by

(2) $$W_{p}\left(\gamma_{i},\tau_{i}\right)={\left[\inf\;E{\left(d\left({Im}_{i}^{G},{Im}_{i}^{T}\right)\right)}^{p}\right]}^{\left(1/p\right)}$$

where E(Im) is the expected value of a random variable Im (estimated impedance in this case), *d* is the Euclidean distance between two points, and the infimum is taken over all joint distributions of the random variables ${Im}_{i}^{G}$ and ${Im}_{i}^{T}$ with PDFs $\gamma_{i}$ and $\tau_{i}$, respectively.

### 5.4. Overhead Evaluation

#### 5.4.1. Area Overhead

The area overhead analysis of FPGA resource usage on SLR0 for various configurations of TDC sensors and inverter-based actuators in ChipletQuake is given in Table 1. The TDC elements consumed minimal resources, using <1% of CLBs and registers, and 10% of URAM each. The actuator chains (e.g., 10,000 LUT1 inverters) significantly increased CLB usage (1.5%) but required no registers or URAM. A complete configuration of ChipletQuake (e.g., eight TDCs + eight actuator elements) increased CLB usage to 15.7% in the SLR, while register utilization remained steady. Moreover, the URAM usage peaked at 80%, mainly due to TDC buffering. We also observed that CLB usage scaled with the number of inverter elements, while register and URAM usage were largely determined by the TDC logic. This demonstrates that actuator chains drive CLB usage, whereas TDCs contribute primarily to register and URAM utilization. Note that it is also possible to decrease the number of elements and inverter density to save on utilization. Furthermore, partial reconfiguration (e.g., Xilinx Dynamic Function Exchange) can be used to free up resources once the sensing process is finished on the verifier side.

#### 5.4.2. Timing Overhead

The total measurement time varied under different configurations of TDC instances, integration times, and frequency sweeps in the ChipletQuake system. Table 2 reports the measurement times by ChipletQuake across the different configurations. The measurement time increased with the number of frequency points, TDC instances, and integration time per frequency. A basic configuration (one TDC, 1 s of integration time, 1 frequency point) completed in under 1 s. Using 20 frequency points and enabling calibration for each of the five captured traces increased the measurement time to 1 min for 10 traces. With two TDCs, 50 frequency points, and 1 s of integration time, the total measurement duration increased to approximately 65 min if 500 traces were collected. A maximum configuration of eight TDCs, 2 s of integration time, and 200 frequency points for 1000 traces required about 18 h to complete. For most of our experimental case studies, we used two TDCs and 20 frequency points, computing the mean of the sensed values across the two TDCs as the distinguisher. Overall, the timing overhead scaled with both the number of frequency points and the number of active TDCs, illustrating a clear trade-off between measurement resolution and runtime. Notably, a significant portion of the total measurement time was due to UART communication delays, as traces were transmitted one by one. To mitigate this bottleneck, one can utilize on-board High-Bandwidth Memory (HBM) or dedicated DRAM to store the traces locally and transfer them over a high-speed interface, such as a PCIe, which is available on the platform.

### 5.5. Profiling Different Hardware Designs

**Figure 9:**
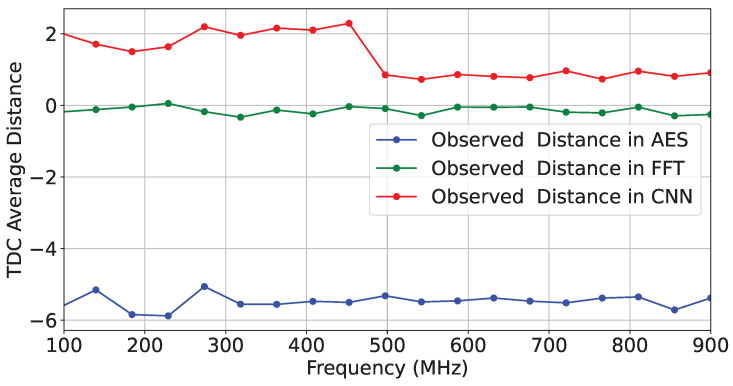
TDC average distance on T=500 traces for different configurations.

For our first set of experiments (Case Study ❶), we collected three sets of traces (T=500) for three hardware applications: AES, FFT, and a simple CNN. We then calculated the average distance between the TDC-sensed values and those from a reference design on SLR1. Figure 9 shows the average distance for each design at the frequency point where the actuators were activated.

### 5.6. Detecting Interposer Tampering

**Figure 10:**
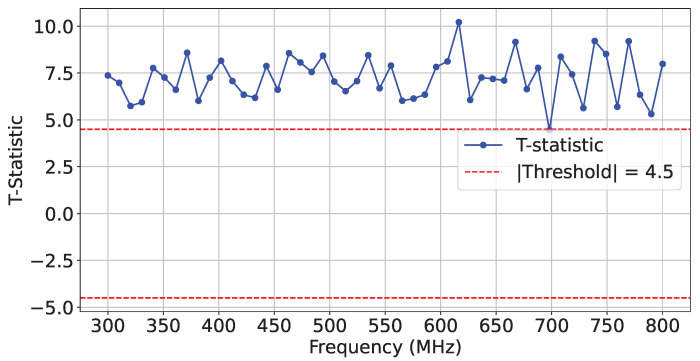
*t*-test on T=1000 traces comparing SLL129 and SLL133 configurations.

**Figure 11:**
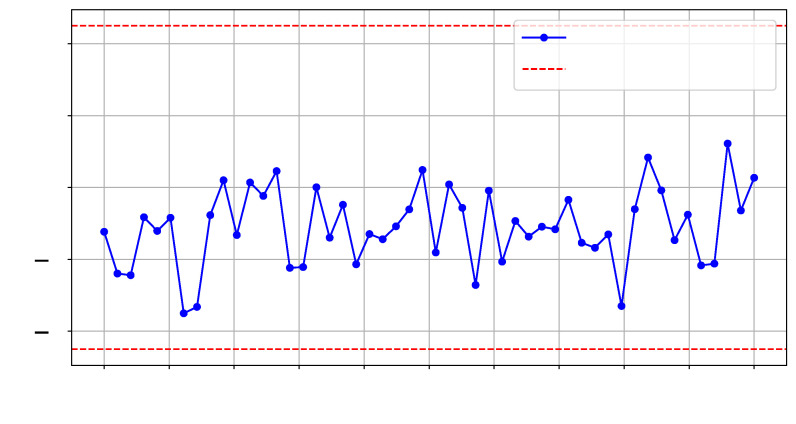
*t*-test on T=1000 traces comparing SLL129 and SLL129-Ref configurations.

To verify the integrity of the interposer in our experiments, we generated two identical logical Register-Transfer-Level (RTL) designs on *SLR 1*, as shown in ❷ in Figure 8. To emulate interposer tampering, we configured a different number of interposer-implemented SLL interconnections in each design, thereby changing the interposer utilization layout. Specifically, our designs employed 129 and 133 SLLs between SLR 0 and SLR 1. Similar to the procedure we used for HT detection, we collected three sets of traces (T=1000 for each set), including reference golden traces. Figure 10 and Figure 11 illustrate the *t*-test scores for each design and the reference reliability scores across the selected frequency range.

**Figure 12:**
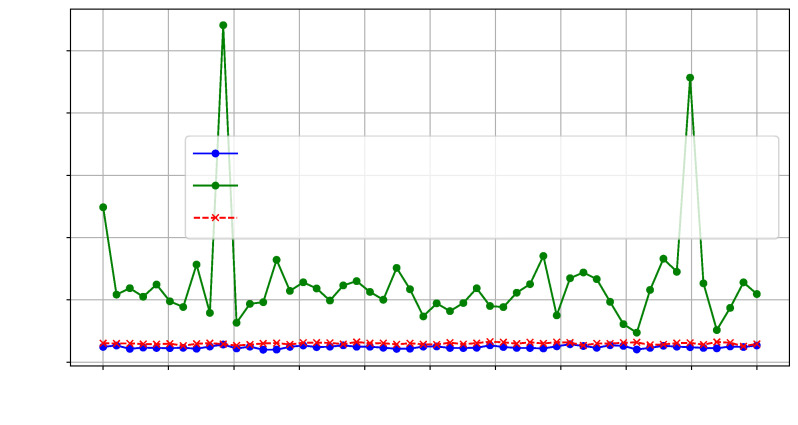
Wasserstein distance on T=1000 traces for AES-SLL129 vs. SLL133.

Furthermore, we calculated a null hypothesis threshold at each frequency point using the Wasserstein distance. For the Wasserstein distance, the null distributions were generated by performing 1000 bootstrap samples from the reference trace set. A significance level of *p*-value = 0.01 was used to determine the threshold for rejecting the null hypothesis. Figure 12 illustrates the Wasserstein distances observed for interposer modifications based on differing SLL utilization.

### 5.7. Footprinting Further SLRs

**Figure 13:**
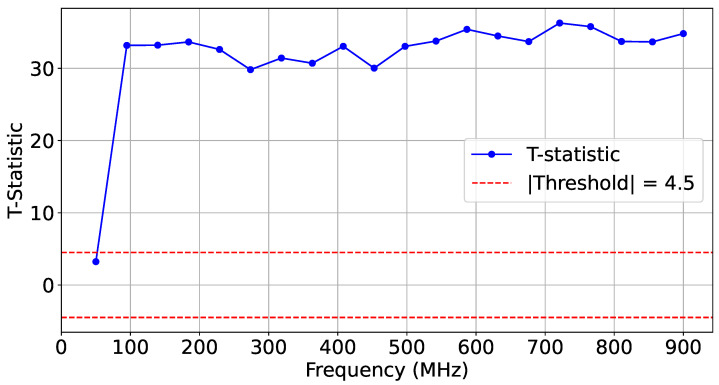
*t*-test on T=500 traces for $config_{1}$ vs. $config_{2}$ at SLR2.

**Figure 14:**
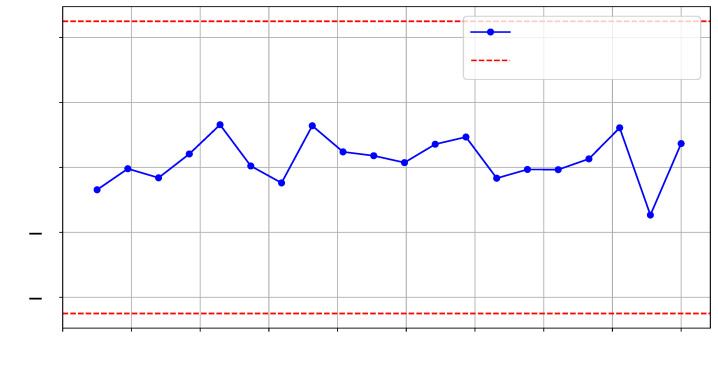
*t*-test on T=500 traces comparing $config_{1}$ and $config_{1}$ -Ref on SLR2.

As illustrated in ❸ in Figure 8, for this experiment, we implemented a test hardware design on SLR2, which is located farther from the verifier deployed on SLR0. We collected two sets of traces (T=500) and an additional reference set, with each set corresponding to the same design in terms of functionality but differing in physical routing on SLR2. We refer to these designs as $config_{1}$ and $config_{2}$. Figure 13 and Figure 14 show the *t*-test scores for each design and the reference reliability scores across the selected frequency range.

**Figure 15:**
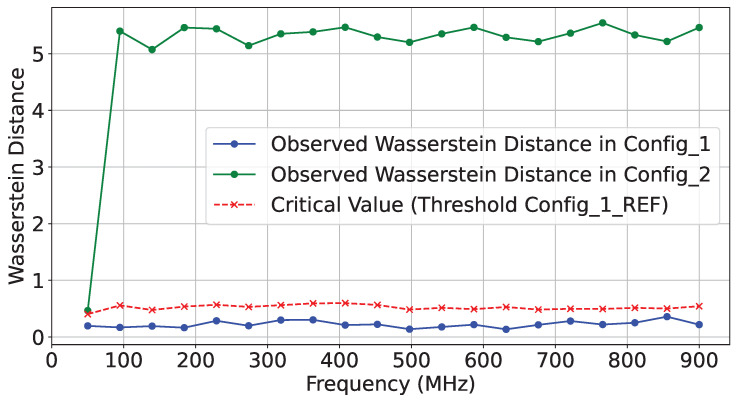
Wasserstein distance on T=500 traces comparing $config_{1}$ and $config_{2}$ on SLR2.

Moreover, Figure 15 shows the Wasserstein distance between $config_{1}$ and $config_{2}$, using $config_{1}$ as the reference.

### 5.8. Detecting Hardware Trojans

In our final set of experiments, as highlighted in ❹, we evaluated *ChipletQuake* against the implementation of hardware Trojans. For this case study, we utilized the RTL HT benchmarks of AES implementations from Trust-Hub [28]. The original HT-free design in these implementations is an AES-128 block cipher IP, featuring an 11-stage pipeline that performs the ten stages of AES encryption on a 128-bit block. For the HT implementation, we used the AES-T1100 variant, which includes an HT payload that modulates AES activity to create a power consumption pattern that leaks the AES key. We deployed each implementation on SLR 1 and performed distinguishability tests from the verifier on SLR 0, powered by ChipletQuake.

**Figure 16:**
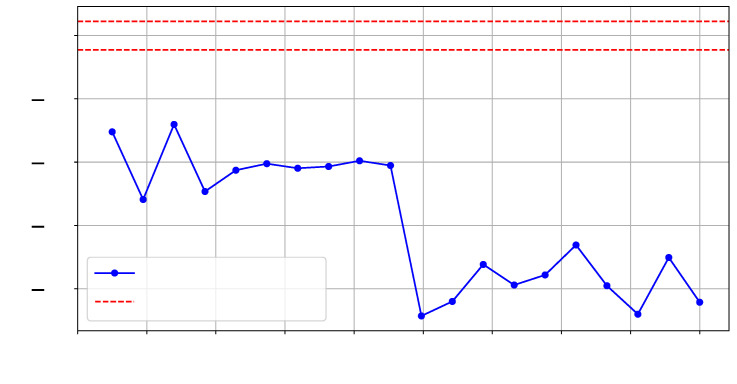
*t*-test on T=500 traces comparing AES-HT-free and AES-HT.

For this experiment, we collected three sets of traces (T=500): a reference set from the legitimate AES_HT_FREE design, a second AES_HT_FREE test set, and a third set from the malicious AES_HT implementation. Figure 16 shows the frequency-based *t*-test distinguishably results captured for two sets of experiments, where AES_HT_FREE and AES_HT were implemented in the adjacent SLR 1.

**Figure 17:**
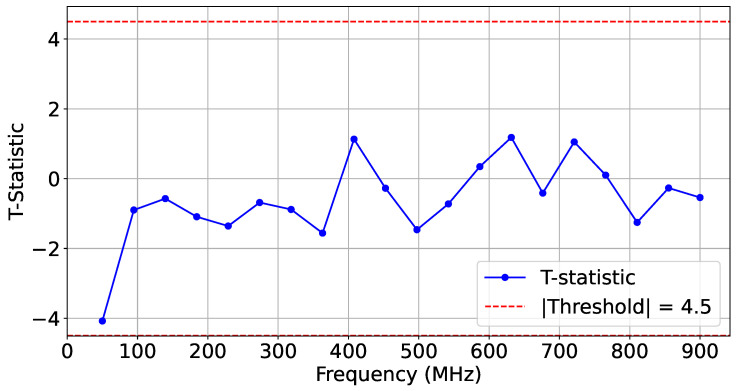
*t*-test on T=500 traces comparing AES-HT-free and AES-HT-free-Ref.

To further showcase reliability, we performed additional HT-free impedance estimation and compared it to the existing HT-free reference traces. Figure 17 depicts the *t*-test scores for two sets of identical implementations of HT-free AES. As expected, the difference scores were confined within the |t|<4.5 similarity range.

**Figure 18:**
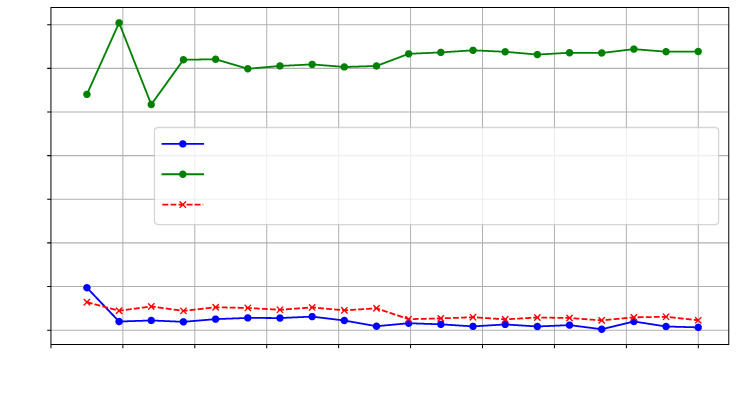
Wasserstein distance on T=500 traces comparing AES-HT-free and. AES-HT.

As shown in Figure 18, the calculated Wasserstein distance for the HT-included IP was significantly higher, indicating that the malicious circuit on the chiplet can be effectively detected.

## 6. Related Works

Power-based side-channel attacks have been shown to be effective in chiplet-based systems for extracting information from adjacent chiplets [29]. Similarly, power-wasting methods (e.g., via ROs) can be used as tools in covert-channel attacks across different SLRs [12]. On the other hand, delay-based sensors can also be designed for use in chiplet fingerprinting. Recently, Mahmod et al. [30] showed that phase shifts in clock signals on Xilinx FPGAs can be accurately measured to identify and fingerprint chiplets for co-location side-channel attacks.

On the defensive side, different approaches have been investigated. The framework proposed by Zhang et al. [6] uses TDC sensors to detect runtime anomalies in adjacent chiplets. Similar works [31,32] have exploited sensed variations in power consumption of neighboring chiplets due to the dynamic execution of target circuits. These works focus on hardware Trojan detection with dynamic monitoring and are only effective upon activation and triggering of the HTs. Hence, dormant malicious circuits (e.g., HTs) cannot be effectively detected unless activated, as the static power consumption of these circuits is extremely low [33,34].

There have also been similar efforts to utilize delay sensors in chiplets for verification and protection in SiPs. Deric et al. [4] introduced a novel framework that deploys interposer-based delay lines as PUFs. The delay variations can then be measured with extremely fine granularity (at the picosecond scale) via phase-shift measurements. Thus, unique signatures can be extracted for each underlying chiplet in the system. As another example, Hoffing [35] used actuator/sensor pairs to fingerprint chiplet-enabled FPGAs in cloud platforms. By energizing and subsequently de-energizing a large load using ROs in a unique pattern, the shared PDN is stimulated, and the resulting transient voltage drop is measured by TDCs as a signature.

A similar actuator/sensor architecture was used by Zhu et al. [8] to estimate impedance via voltage fluctuations and time-to-frequency conversion using FFT. Although Zhu et al. proposed a novel RO-based power-wasting circuit enabled by specific patterns to stimulate different spectral bands, this potentially reduces the achievable SNR for intra-SLR fingerprinting.

Despite these methods, our results indicate that intra-chip fingerprinting via impedance analysis requires high-frequency stimulation. In contrast to prior works where static [35] or low-bandwidth [8] stimulation was used, our framework stimulates the power delivery network at frequencies above 100 MHz and up to 1 GHz, enabling the realization of RTL-level fingerprinting.

Table 3 summarizes existing chiplet verification frameworks and compares them with ChipletQuake.

Our work presents a frequency-domain sweeping approach that yields a rich impedance profile, which can be processed to identify static circuits that do not need to be dynamically activated. This methodology offers sufficient accuracy to detect tiny, static, dormant HTs.

## 7. Discussion

### 7.1. Limitations

Frequency stimulation for an embedded VNA typically requires either integrating analog components within the device or leveraging existing clock generators on the chip. ChipletQuake approximates sinusoidal waveforms directly using source clock signals, making it well-suited for differential measurement applications such as hardware Trojan and tamper detection. However, in non-differential scenarios, this approximation may introduce non-trivial errors. To address this limitation, one approach is to design programmable load circuitry capable of emulating sine-like power patterns [36]. Nonetheless, the maximum achievable frequency of such a programmable sine generator is constrained by the highest frequency supported by the on-chip clock and PLL components. Techniques like phase-shifted PLL-based waveform generators [37] may offer a path forward, enabling higher actuation frequencies suitable for ultra-high-frequency impedance sensing.

Another important challenge is the scalability of area coverage, which warrants further experimental investigation. As observed in our experiments, the SNR of measurements obtained from the embedded VNA is higher in chiplets located near the source-side SLRs compared to those farther away. Although the ChipletQuake verifier framework can be modularly deployed in any chiplet (i.e., SLR in this experiment), capturing reliable measurements from distant target chiplets can be problematic. This is primarily due to the attenuation of the actuated signals and the reduced ability to detect picosecond-level variations. One potential avenue for improvement involves increasing the size of the actuators and expanding the sample size of the collected traces to enhance measurement fidelity. This indeed comes with much more power and time overhead for the detection procedure.

While our initial experiments were conducted under controlled laboratory conditions, it is important to acknowledge the limitations this imposes on generalizability in terms of temperature, voltage, and electromagnetic interference (EMI). Notably, the ChipletQuake framework relies on differential measurements, which inherently suppress the low-frequency fluctuations caused by these variations or other external physical parameters. In addition, the system employs dynamic calibration at regular intervals (e.g., every N traces), which helps mitigate temporal drift and improves consistency over time. Despite these measures, potential sources of fast and burst noise, such as thermal gradients and EMI, still influence the system in uncontrolled environments. To address this, we emphasize the use of peripheral sensors (e.g., temperature sensors) to tag each measurement with contextual metadata. These tags facilitate detection strategies that are aligned with test-time environmental conditions. Nevertheless, future work should explore more comprehensive temperature-controlled and EMI-exposed testing to evaluate the framework’s robustness in realistic deployment scenarios.

### 7.2. Further Applications and Improvements

While this work demonstrates the feasibility of using ChipletQuake for fine-grained impedance sensing and verification, future directions include the development of a full challenge–response protocol for hardware authentication. As discussed in Section 3.2, the large configuration space of the system, comprising selectable actuator elements, frequency points, and sensor positions, can be leveraged to construct dynamic and unique verification challenges. By varying these parameters across different verification sessions, one can improve both the unpredictability and the security of the signature, making it more resistant to spoofing or tampering.

Although this paper presents only the baseline case where all actuator elements and frequency points are used and only TDC sensors are measured, we envision a protocol in which subsets of frequencies and sensor locations are randomly selected to serve as a secure cryptographic protocol. A critical next step is to evaluate how such selective excitation and sensing configurations impact the reliability and stability of the golden signature across repeated measurements and under adversarial perturbations. We identify this as a promising area for future exploration toward practical and secure chiplet-level attestation. Moreover, the same methodology can be extended to other FPGA platforms and possibly other chiplet-enabled platforms in future research.

It might be possible to use this framework to perform dynamic verifications as well. This means that the signature procedure can be performed during the switching activities of the adjacent chiplet. Impedance-based dynamic fingerprinting potentially opens up the possibility of carrying out side-channel attacks on the neighboring chiplets. Furthermore, as another application, it is also possible to perform extensive templating/profiling to fingerprint a target IP in threat models where physical side channels are considered.

Furthermore, considering the timing overhead in scenarios with low SNR, where a large number of traces are required, a potential improvement could be achieved by utilizing HBM instead of UltraRAM on the FPGA fabric. Leveraging HBM significantly reduces host-to-target communication, as it offers substantially larger on-chip memory capacity compared to UltraRAM.

## 8. Conclusions

The transition to chiplet-based designs not only addresses critical challenges in modern semiconductor manufacturing but also introduces significant security vulnerabilities in the hardware supply chain. This paper presented ChipletQuake, a framework for post-silicon verification of physical security in chiplet-based systems. By leveraging impedance sensing of the power delivery network, ChipletQuake effectively detects tampering events in the interposer and neighboring chiplets without requiring additional hardware. The impedance estimation method is capable of detecting dormant, static, and subtle modifications in the MCMs. Our experimental results demonstrate its capability to identify hardware Trojans and interposer tampering, validating its effectiveness in enhancing the security of FPGA-based chiplet systems.

## Figures and Tables

**Table 1:** Detailed area overhead breakdown of different configurations of ChipletQuake.

TDC Inst	Inv Chain Inst	CLB			Register		BRAM	SLR Util%		
		**LUT1**	**LUT5**	**CARRY8**	**FDRE**	**LDCE**	**URAM288**	**CLBs**	**Regs**	**URAM**
1	0	0	165	16	765	32	32	0.58	0.09	10.0
0	1	10,001	0	0	0	0	0	1.51	0.0	0.0
8	8	80,008	1342	136	4841	256	288	15.71	0.57	80.0
8	32	320,032	1347	136	4881	256	288	50.99	0.57	80.0

**Table 2:** Time overhead analysis of different configurations of ChipletQuake for evaluation.

TDC Inst	Integration Time (s)	Frequency Points	Calibration Freq.	Differential Meas.	Trace Num.	Meas. Time (s)
1	1	1	1	No	1	0.760 (<0 min)
1	2	20	5	Yes	10	62.6 (∼1 min)
2	1	50	5	Yes	500	3881.2 (∼65 min)
8	2	200	5	Yes	1000	62,099.2 (∼18 h)

**Table 3:** Comparison of existing chiplet verification frameworks.

Framework	Target Platform	Methodology	Actuators	Sensors	Overhead	Application
Delay-based PUF [4]	Virtex UltraScales+ FPGA	Interposer Delay PUF	N/A	Clock Phase Shift	Very Low	Chiplet Fingerprinting
SiPGuard [6]	ARM MPS3 FPGA	ML-based Detection	N/A	TDC	Medium	Detecting HW Trojan Activation
PDNSig [8]	Virtex UltraScales+ FPGA	PDN Impedance Estimation	RO	TDC	Low	Chiplet Fingerprinting
CHSM/CSIP [31,32]	ARM MPS3 FPGA	Dedicated Monitoring Chiplet	N/A	TDC	High	Detecting HW Trojan Activation
ChipletQuake (Ours)	Virtex UltraScales+ FPGA	Impedance Sensing	Frequency Sweeping Inv Chain	TDC Array	Adjustable (Medium)	Detecting Dormant/Inactive HW Trojans

## Data Availability

The original contributions presented in this study are included in the article. Further inquiries can be directed to the corresponding author.
